# Putting the “Joy” in joint attention: affective-gestural synchrony by parents who point for their babies

**DOI:** 10.3389/fpsyg.2014.00879

**Published:** 2014-08-12

**Authors:** David A. Leavens, Jo Sansone, Anna Burfield, Sian Lightfoot, Stefanie O’Hara, Brenda K. Todd

**Affiliations:** ^1^School of Psychology, University of Sussex, East SussexUK; ^2^Department of Psychology, City University LondonLondon, UK

**Keywords:** pointing, smiling, embodied cognition, intersubjectivity, affective -gestural synchrony

## Abstract

Despite a growing body of work examining the expression of infants’ positive emotion in joint attention contexts, few studies have examined the moment-by-moment dynamics of emotional signaling by adults interacting with babies in these contexts. We invited 73 parents of infants (three fathers) to our laboratory, comprising parent-infant dyads with babies at 6 (*n* = 15), 9 (*n* = 15), 12 (*n* = 15), 15 (*n* = 14), and 18 (*n* = 14) months of age. Parents were asked to sit in a chair centered on the long axis of a room and to point to distant dolls (2.5 m) when the dolls were animated, while holding their children in their laps. We found that parents displayed the highest levels of smiling at the same time that they pointed, thus demonstrating affective/referential synchrony in their infant-directed communication. There were no discernable differences in this pattern among parents with children of different ages. Thus, parents spontaneously encapsulated episodes of joint attention with positive emotion.

## INTRODUCTION

Joint attention is the ability to capture and re-direct the attention of a social partner, and to follow another’s communicative cues to a specific locus (e.g., Moore and Dunham, 1995; Bard and Leavens, 2009; Leavens and Racine, 2009; Seemann, 2012). Joint attention refers to a suite of triadic communicative skills that typically develop in humans and apes late in their infancy periods, near the end of the first year of life, and includes such behavioral developments as pointing, following the pointing and gaze direction of others, using emotional information from a caregiver to regulate one’s response to novel objects (social referencing), and other tactics involving the coordination of attention to a common focus (e.g., Carpenter et al., 1998; Butterworth, 2001; Striano and Bertin, 2005; Racine and Carpendale, 2007; Bard et al., 2014). Under existing strictures in some contemporary psychological theories, this kind of coordination requires that babies develop a reasoning capacity, based on an ability to represent the invisible contents of others’ minds; pre-verbal human babies are held to point to things because they can represent the perceptions, even knowledge, of their social partners and wish to manipulate those perceptions and those knowledge states (see, e.g., Racine and Carpendale, 2007, for a review and critique).

Joint attention in human infants occurs in social contexts characterized by dynamically changing emotional contours. There is a growing body of work examining the dynamic expression of infants’ positive emotion in joint attention contexts (e.g., Adamson and Bakeman, 1985; Hobson, 1993; Messinger and Fogel, 1998; Jones and Hong, 2001, 2005; Reddy, 2001, 2003; Striano and Bertin, 2005; Carpenter and Liebal, 2012). For example Adamson and Bakeman (1985) reported high rates of positive affect when infants from 6–18 months of age were jointly engaged around objects with their mothers. Jones and Hong (2001) reported that, late in the first year of life, infants begin to incorporate their own smiling behavior into intentional communication with their mothers (and see Jones and Hong, 2005). Reddy (2001, 2003) outlined a developmental pathway into triadic reference grounded in infants’ experiences of themselves as objects of attention and intentional actions. In particular, Reddy’s account specifies the affective qualities manifested during infants’ early engagements with others as a field of experience that can be generalized to objects later in the first year of life. Recently, Carpenter and Liebal (2012) have described, in conceptual terms, the role of mutual visual regard with positive affect between babies and their parents as a kind of acknowledgment of the mutual awareness of the jointness of the interactions, the idea being that babies and their mothers acknowledge the shared nature of these joint attention episodes with mutual gaze and smiles. These findings and conceptual advancements were presaged by Hobson (1993), who speculated that “the development of a child’s awareness of propositional attitudes might begin with more or less direct perception of other people’s affective attitudes” (p. 240). Thus, according to Hobson, affective awareness scaffolds or bridges later-developing conceptions of mental attitudes. Few studies, however, have examined the moment-by-moment dynamics of emotional signaling by adults interacting with babies in these triadic contexts. These affective landscapes may have significant bearing on infants’ motivations to follow into another person’s focus of attention, for example, following their pointing gestures or their line of regard.

The present study was originally designed by Leavens and Todd to examine parents’ coordination of the hands that they used to point to distant dolls arranged in an arc and to support their children in their laps, the question being at what angular displacement to left or right would parents switch the hands being used to physically support their babies in their laps and being used to point (Todd and Leavens, in preparation)? Upon initial examination of the videotaped footage, it seemed to be the case that the parents were marking their own pointing gestures with bursts of positive emotion. This has significant bearing on a longstanding debate in developmental psychology: are human children evolutionarily prepared for engaging in joint attention, as argued by Tomasello et al. (2007), or do parents shape infants’ attention-oriented behavior with social reinforcement, as long argued by Moore (e.g., Moore and Corkum, 1994)?

In considering the different predictions of these two classes of theory, we reasoned, following Leudar and Costall (2004), that nativist accounts like that of Tomasello et al. (2007) assume that there is an epistemological gap between a communicative behavior and its psychological underpinnings; i.e., there is a theoretical commitment to the idea that invisible psychological processes cause communicative behavior, and it is the role of the developing infant to discover this relationship (see also Leavens et al., 2005; Froese and Leavens, 2014, for discussions of this issue). As a consequence of this embedded assumption, external features of the ontogenetic contexts in which children develop their social skills are assumed to be typical for the species. Therefore, we could not specify any pattern of behavior, in advance, that could falsify a theoretical claim of evolutionary preparedness for joint attention in humans (see also Bard and Leavens, 2014, for a review of theoretical positions that omit developmental experience as an explanatory factor in the development of social skills; also see Bateson, 1972; Churcher and Scaife, 1982; Zukow-Goldring, 1997). In contrast, learning- or experience-based accounts of the development of joint attention do make some global predictions about the patterns of reward in the lived experiences of children who are developing these skills (see, esp., Moore and Corkum, 1994; Reddy, 2003). In particular, if children are to learn to attend to deictic signals, then there must be some way that these physical acts are marked as being, somehow, special-in-relation-to external objects and events.

Accordingly, we set out to characterize the smiling behavior of the parents in this study in temporal relation to key events in each of trials: at several time points before the doll was animated, at the moment the doll was activated, at the moment of maximum extension of the parents’ pointing hands, at the moment the pointing hand was maximally retracted, the moment the doll’s activation ceased, and at two subsequent time points. Our reasoning was that if parental smiling behavior was paired with their referential signals (their points), then this would provide evidence relevant to at least two broad classes of theoretical axioms: first, as a kind of affective-referential precursor to the affective-conceptual links described by Hobson (1993) and Reddy (2001, 2003) and, second, as a pattern of contingencies in social reward that could, in principle, exert the kind of socially grounded attention-shaping processes required by Moore and Corkum’s (1994) theory. Alternatively, if we did not find a close temporal association between pointing and smiling behavior, this would have some bearing on the generality of developmental process models grounded in environmental factors, like social reinforcement; in other words, because learning models require contingent social reinforcement, the present study comprises a direct test of the hypothesis of socially based reward contingencies in parent-infant interaction.

Of particular relevance to the emerging science of embodied intersubjectivity is that the interactive phenomena we describe here comprise bodily manifestations of the interactive accompaniments to pointing; thus, this experimental context is an ideal test bed for exploring behavioral coordination in intersubjective activities. As Froese (2011) recently emphasized, the rapidly emerging strands of embodied approaches to understanding cognitive development, including enactivist and dynamic systems theories, markedly expand the kinds of questions we can ask about intersubjective engagement (e.g., Zukow-Goldring, 1997; De Jaegher and Di Paolo, 2007; Wilson and Golonka, 2013). The present context, in which parents point for their young children, is ideal for examining the bodily vehicles of attention scaffolding.

## MATERIALS AND METHODS

### PARTICIPANTS

We recruited parents with young babies through advertising posters with tear-off cards on which were printed the contact details of the infant study unit, at the University of Sussex. We invited 76 parents of infants (3 fathers) to our laboratory, of whom 73 completed testing, comprising parent-infant dyads with babies at 6 (*n* = 15), 9 (*n* = 15), 12 (*n* = 15), 15 (*n* = 14), and 18 (*n* = 14) months of age (two of the three remaining dyads were excluded due to infant fussiness, and one because of experimenter error—specifically, the videotape was accidently overwritten).

### PROCEDURE

**FIGURE 1:**
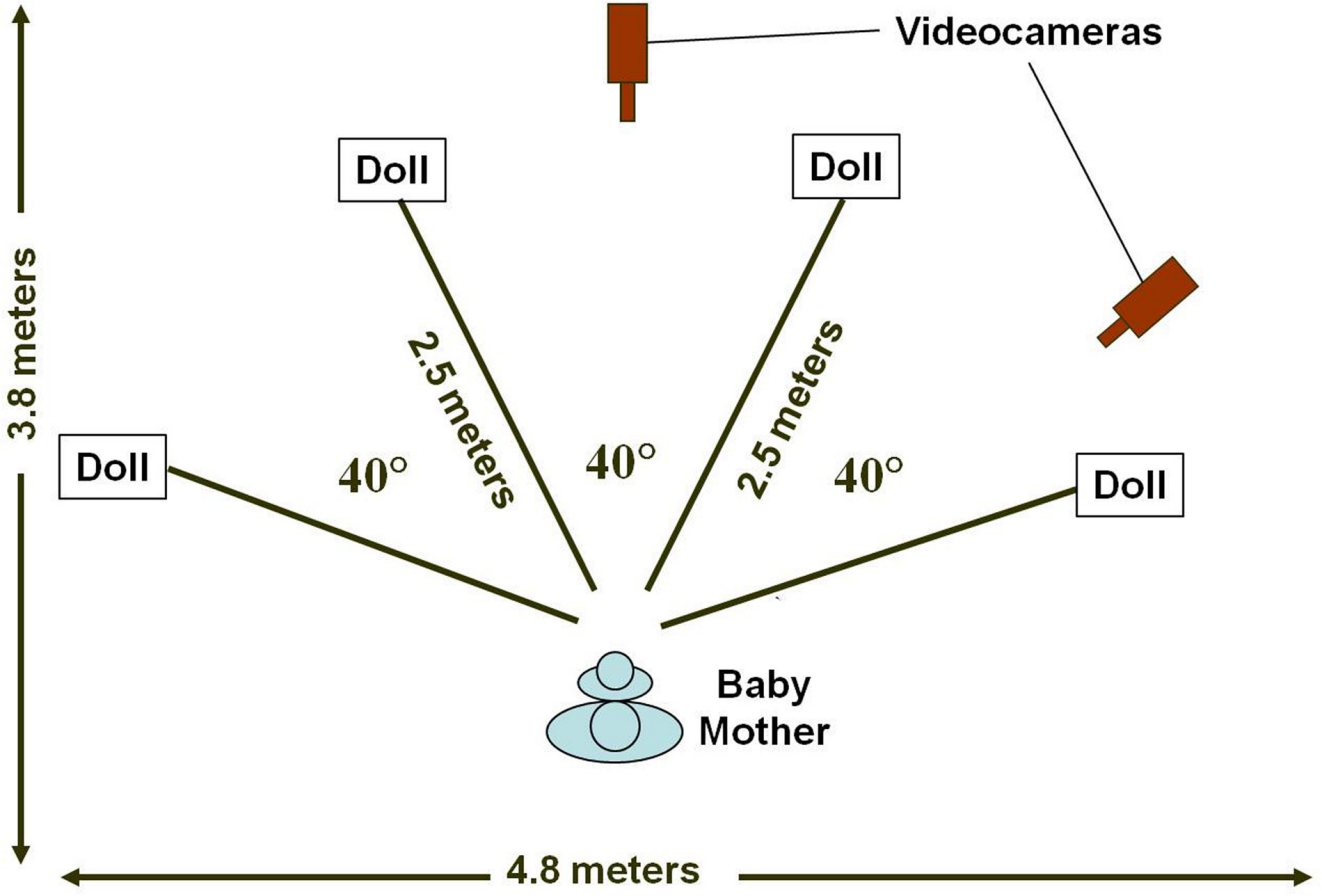
**Schematic of the experimental setup.** Drawing not to scale.

Parents were asked to sit in a chair centered on the long axis of a 5 × 4 m room with symmetrical illumination and a beige curtained backdrop (**Figure 1**). The parents held the children in their laps. Four mechanical dolls were arranged in an arc around the dyads, 2.5 m from their chair, at symmetrical angular displacements of 20 and 60° to the left and right of their midlines. Two video cameras were placed, respectively, centrally and 45° to the right of the dyads; images were mixed to a split screen and this split screen image was recorded on Super VHS video. Dyads were randomly assigned to random sequences of doll activation so that each of the four dolls were animated on the first four trials and then this same sequence was repeated for an additional four trials, rendering eight trials per dyad. As each of the dolls was animated from a control room adjacent to the laboratory, its “arms” and “legs” oscillated up and down while auditory signals (a recorded female voice repeating the phrase, “Hey, baby!”) were emitted from a speaker mounted behind each doll’s ”head” for a duration of 5 s. Parents were asked to point to the dolls when they were animated. No other specific instructions were given.

### CODING AND ANALYSES

The onsets of eight 1-s intervals were defined for each trial: (a) 6 s prior to doll activation, (b) 3 s prior to doll activation, (c) the instant the doll was activated, (d) the time at which the maximum extent of parents’ points were displayed, (e) the time at which the pointing hand was maximally retracted (note that in every observation, the hand used to point was retracted and brought to a clear, unambiguous resting position), (f) the moment the doll was inactivated (5 s after doll activation), (g) 3 s after the doll was inactivated, and (h) 6 s after the doll was inactivated. On each of the eight trials, for each of these eight 1-s intervals, parents were dichotomously classified as either “smiling” or “not smiling” during that 1-s interval. On 27% of the 4,672 observation intervals it was not possible to see the faces of the parents, so the dependent variable was the proportion of trials across the eight intervals in which the parents smiled, including only intervals in which the parents’ faces were clearly visible. Because some infants became fussy, the total number of trials per parent–infant dyad ranged from 4–8 (we included all dyads that had completed at least four trials). Because not every parent–child dyad participated in the same number of trials, the dependent variable was the proportion of trials in which parents smiled.

Two coding teams, each comprising independent pairs of researchers, performed separate passes through the entire corpus, each team coding to a consensus. Because, initially, we were interested in characterizing the intensity of smiling on each trial, the first coding team (Burfield and O’Hara) applied a three-category scheme to only some of the observational intervals described above: parents were categorized as (a) not smiling, (b) weakly smiling, and (c) strongly smiling. We found it difficult, however, to define the boundary between weakly smiling and strongly smiling, in objective terms, to the second coding team (Lightfoot and Sansone). Therefore, the second coding team scored an expanded number of intervals, dichotomously classifying parents as either (a) not smiling, or (b) smiling, as described above; this was the data used for the analyses reported here. Smiles were coded when the corners of the mouth could be seen to be raised (Ekman and Friesen, 1978). Due to a late-discovered technical problem with the microphone, few recorded video clips contain audible speech; this was not considered to be problematic with respect to the original hypotheses the study was designed to test, pertaining to cradling and handedness, but it does constrain our present analyses and conclusions entirely to visual information.

### RELIABILITY

As noted above, there were two coding passes through the data, using slightly different coding schemes. For purposes of reliability assessment, we collapsed the initial coding of weakly smiling and strongly smiling into a single category of “smiling” and then directly compared these data with the inherently dichotomous data of the second coding team. Reliability was assessed as the agreement on parental smiling in intervals coded by both teams (25% of the corpus) Cohen’s κ = 0.64. Because the probability of a 1-s interval being coded as either smiling or not was highly variable across intervals, and because the coding system was very simple, therefore this is a very good level of agreement (see discussion in Bakeman and Quera, 2011, pp. 65–68). Landis and Koch (1977) characterized κ values between 0.61 and 0.80 as “substantial agreement” (page 165).

## RESULTS

**FIGURE 2:**
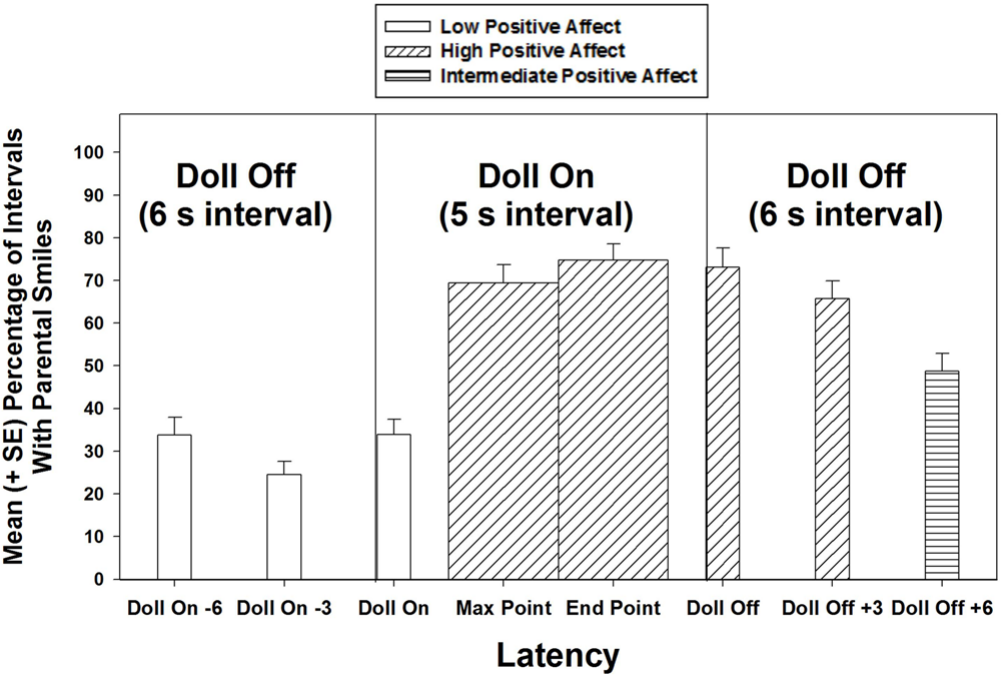
**Parents exhibited low positive affect until they pointed, at which time they exhibited high positive emotion.** By 6 s after the doll had been turned off, they exhibited moderate levels of positive emotion. These three levels of positive affect were determined by exhaustive Bonferroni-corrected, pairwise tests.

An 8 (intervals) × 5 (age group) mixed ANOVA revealed that parents smiled non-randomly throughout the experimental trials, *F*(7,476) = 55.67, *p* < 0.001. Systematic pairwise comparisons, with Bonferroni corrections for multiple tests revealed a general pattern of three “levels” of parental smiling: from a LOW level of smiling at all time points preceding the doll activation up to the moment of activation (i.e., the first three time points in the trials), through an epoch of a HIGH level of smiling starting from the maximum extension of the parental points and ending 3 s after the doll had stopped moving (i.e., the next four time points in the trials), and, finally, an INTERMEDIATE level of smiling at the last time point measured in each trial, 6 s after the doll’s animation ceased, as the smiling returned to baseline levels (see **Figure 2**; **Table 1**). In other words, levels of parental smiling within the “HIGH” level did not differ statistically in pairwise comparisons, but they did differ from smiling levels in INTERMEDIATE and LOW, and this pattern held for all three levels of smiling, with only one exception: within the LOW category, smiling during the second interval, DOLL ON -3 s, was statistically lower than both of the immediate adjacent levels, also labeled LOW, but stood in an identical relation with these adjacent intervals to all intervals labeled HIGH and INTERMEDIATE (i.e., there was statistically less smiling in all intervals labeled LOW, compared to INTERMEDIATE and HIGH smiling levels). Because our minimum intertrial interval was 12 s in duration, we could not extend our observations later in time during each trial, because this would have overlapped with successive trials in many instances; this is why our analyses do not capture a full return to baseline levels by the end of the trials. Thus, parents encapsulated these episodes of joint attention, in which they pointed to distant targets, with an envelope of positive emotion.

**Table 1:** Percentage of parents who smiled, by trial, at eight time points within each trial.

Interval									
Trial no.	Doll on -6 s	Doll on -3 s	Doll on 0 s	Max. point	End point	Doll off 0 s	Doll off +3 s	Doll off +6 s	Mean of intervals (SD)
1	26	**22**	27	66	**89**	72	68	54	53 (25)
2	41	**24**	33	68	**87**	82	71	50	57 (23)
3	38	**32**	38	80	74	**85**	67	49	58 (21)
4	35	**16**	35	**74**	71	**74**	58	38	50 (22)
5	29	**18**	32	68	**80**	74	67	53	53 (23)
6	32	**24**	31	62	68	**73**	61	43	49 (19)
7	**28**	29	38	**76**	67	70	54	41	50 (19)
8	23	**17**	29	**76**	71	68	68	43	49 (24)

Mean of trials (SD)	31 (6)	**23** (6)	33 (4)	71 (6)	**76** (9)	75 (6)	64 (6)	46 (6)	–

*The number of parents whose faces were visible at any given 1 s-interval, on any given trial, ranged from 38–69, mean = 52. Values in parentheses are standard deviations. Tabled values in bold are the minimum and maximum values in each row (ties are both bolded). Percents reported exclude parents whose faces were not visible.*

**FIGURE 3:**
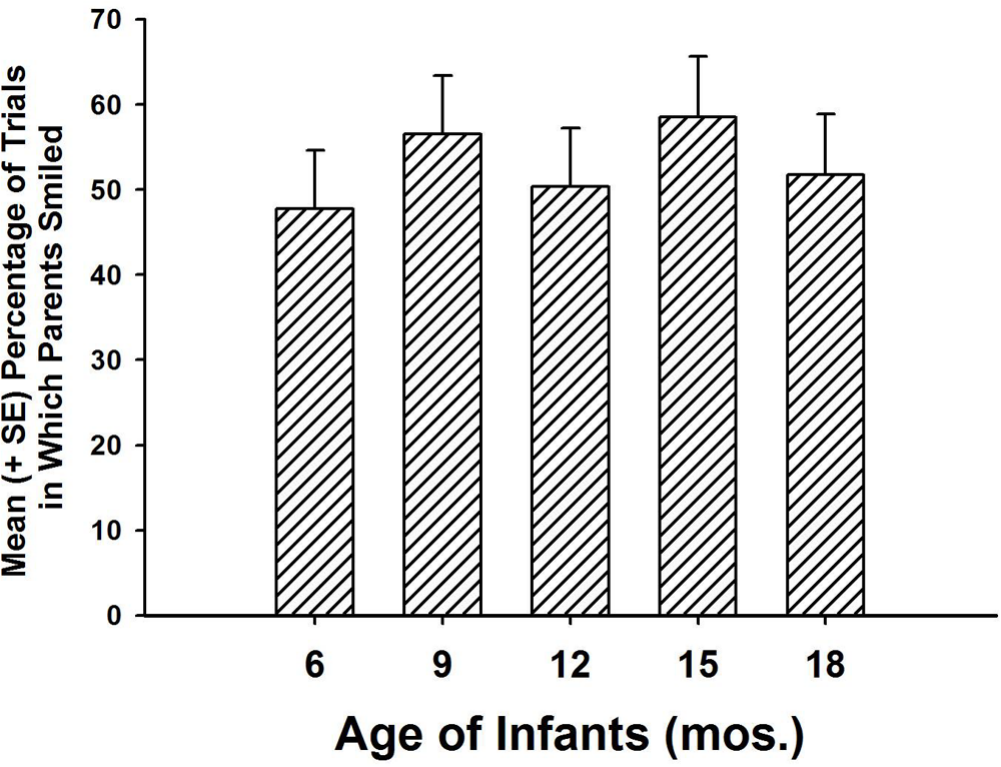
**Parents did not differ in the amount of smiling they displayed for their infants, across the entire age range of this study, with infants from 6 to 18 months of age**.

There was no influence of age group, *F*(4,68) = 0.41, *p* = 0.799, nor was there an interaction between interval and age group, *F*(28,476) = 1.18, *p* = 0.242 (see **Figure 3**). Parents encapsulated joint attention episodes with positive emotion across the entire age range of our infant subjects, from 6 to 18 months of age. There was modest, but statistically significant variability in the number of intervals in which parents smiled across trials (Greenhouse–Geisser corrected *F*(4.42, 317.86) = 55.91, *p* < 0.001. To determine whether there was any evidence of parental habituation in smiling to the doll animations, we summed the number of intervals in which parents smiled in the first four trials and compared this to the number of smiles in the last four trials, finding that there was a significant difference [paired samples *t*(72)= -2.09, *p* = 0.041]. However, there were more intervals with smiling in the second half of the experiment (mean = 13.3, SD = 8.9) than in the first half of the experiment (mean = 12.1, SD = 7.1), indicating that, if anything, the experiment elicited more smiling with the passage of time.

We found no influence of infant birth order on parental smiling behavior [*F*(2,70) = 1.41, *p* = 0.252]), nor did we find a relationship between parental age and smiling behavior (Pearson’s *r* = 0.05, *p* = 0.681). Finally, parents did not smile differentially as a function of infant gender: *t*(71) = -0.47, *p* = 0.642.

## DISCUSSION

There are two substantive findings from this study of 73 parent-infant dyads. First, parents displayed peak positive emotion, as evidenced by smiling behavior, that was temporally synchronized with their pointing gestures and their immediate aftermaths. Second, this pattern characterized the entire sample of children from 6–18 months of age. This distinctive pattern of positive emotional display while pointing to entities has significant relevance for contemporary theoretical interpretations of infant pointing. The dominant, internalist (or telementational – see the detailed analysis and critique of internalist theories of development by Leudar and Costall, 2004) perspective on human communicative development interprets infants’ abilities to triangulate with others on a common focus as evidence for infants’ developing abilities to represent the abstract visual perspective of others, along with the developing appreciation of others as psychological entities (e.g., Povinelli et al., 1997; Tomasello et al., 2007). Thus, in mainstream cognitive psychology, there is, arguably, an overweening concern with computational models of human cognitive development; or as Shotter and Newson (1982, p. 37) put it: “[t]raditional cognitive psychology has now set its sights upon discoving the nature of the ‘inner computer’ … people use in achieving their actions.” We think that our findings draw attention to the external, ecological features of the communicative environments in which children necessarily construct their habits of response to the communicative bids of their caregivers.

This synchronization of parents’ positive emotional signaling at the peak extensions of their own pointing gestures highlights the environmentally situated placement of key affective information about the nature of these joint attention interactions. This pattern raises the possibility that, in accordance with the analyses of Moore and Corkum (1994), Zukow-Goldring (1997), and Rader and Zukow-Goldring (2012), parents may actively, if apparently unconsciously, shape the attention-deployment patterns of their children, at least in some cultural contexts. If these patterns of parental affective signaling do exert an influence on the development of infants’ responses in joint attentional social frames, then we would predict substantial cross-cultural variation in these developmental profiles (Schieffelin and Ochs, 1989; Triesch et al., 2006; Keller, 2007, 2012). Although there is not a lot of directly relevant literature, what evidence exists is consistent with the idea that both the amount of time parents spend in coordinated joint engagement around objects with their babies and the emotional tones of those interactions differ substantially across settings. For example, Bakeman et al. (1990) reported that aboriginal !Kung infants spent only 1.6% of observed intervals engaged in joint object involvement, compared (with some qualifications) with a North American sample (Clarke-Stewart, 1973), in which about 4.5% of intervals involved joint object involvement between babies and their mothers. Abels et al. (2005) reported relatively low levels of joint object involvement between mothers and their babies in both rural and urban settings in a study from India. Vogt and Martin (2013) reported substantially fewer co-speech gestures by parents of young children in rural Mozambique communities, compared with urban communities in Mozambique. Salomo and Liszkowski (2013) observed that Mayan babies pointed with their index fingers at much reduced rates, compared to both Dutch and Chinese children, and also spent significantly less time in triadic joint action than Dutch and Chinese children. Thus, cross-cultural differences in the incidence of object-centered joint engagement are well-established, and the present findings suggest that these differences may be accompanied by cross-cultural differences in maternal affective tone in relation to object-centered coordination of attention.

The absence from the present study of any apparent influence of infant age on parental smiling behavior suggests that this pattern of gestural/affective synchrony may characterize intersubjectivity across a wide swathe of infancy and infant competencies. Parents of even our youngest infants (6 months of age) still smiled most frequently at the peak of their pointing gestures. There is little evidence of point- or gaze-following ability in Western children at this age (e.g., Butterworth and Grover, 1988; Butterworth, 2001; Deák et al., 2008), so if parents are displaying this pattern of pairing high positive affect with pointing gestures outside of the laboratory, then this could provide a stable emotional contingency contour around parent-initiated joint attention long before babies evidently can use these kinds of referential signals and continuing well into the second year of life. In other words, if these patterns of affective/referential synchrony are manifested in the home environments of these babies, then both the babies’ attentional deployments and their attitudes about novel objects or events may be developmentally shaped into a typical Western pattern of joint object involvement (see, e.g., Moore and Corkum, 1994; Rossmanith et al., in press). Learning- and ecologically based theoretical accounts of the development of joint attention ability in humans, like those of Shotter and Newson (1982), Moore and Corkum (1994), and Triesch et al. (2006) require this kind of stability in these contingent social reward. Thus, the present study, despite its *a posteriori* approach, was sufficiently powerful in design to have significantly challenged learning-based accounts of sociocognitive development, by failing to find either (a) that parents did not pair their referential gestures with smiles or (b) that parents only displayed these patterns for a minority of our age groups. In accordance with environmentally oriented theoretical accounts, the parents in this study paired their own pointing gestures with smiles across the entire age range of our sample, with infants from 6 to 18 months of age. If the present findings can be extended to the rearing environments of children with their families, outside a laboratory context, then these data suggest that affective-referential synchrony might occur across a vast swathe of human infancy, at least in Western, post-industrial cultural environments. Thus, joint attention in humans is situated in a social landscape of emotional markers for key intersubjective experiences (Churcher and Scaife, 1982; Shotter and Newson, 1982). Churcher and Scaife (1982), for example, noted that children learning to follow the gaze and pointing cues of their caregivers may be motivated not only by the potentially rewarding sight of the indicated entity, but also the “social reactions” of their caregivers (p. 127; see Bard et al., 2014, for evidence of the association between affect and joint attention in infant chimpanzees). Shotter and Newson (1982) noted that a human child, “although perceptually distinguishable from her environment as an individual … is not as such physically isolable from it; she exists (as an open system) only in mutual relation to it” (p. 34). Thus, our findings are consistent with developmental accounts that emphasize the non-computational, distributed concomitants of joint attention, insofar as these babies’ social environments displayed distinctive envelopes of dynamic changes in the expression of positive emotion, peaking at the time of parents’ pointing gesture extensions.

In contrast, nativist accounts of the development of joint attention in human children, such as those by Butterworth (2003), Povinelli et al. (2003), and Tomasello et al. (2007) all posit a species-unique human specialization for triadic joint engagement, based on hypothetical cognitive and/or motivational capabilities that are also allegedly unique to our species. What holds these disparate nativist perspectives together as a class of theoretical speculation is the postulate that human capacities to follow into and to direct the attention of others are predicated on evolutionary adaptations of cognitive and/or motivational systems in our lineage, and shared by all extant humans. As Racine (2012; and see, e.g., Racine and Carpendale, 2007) has pointed out, the hypothesis of a human biological adaptation for joint attention is necessarily an assumption without empirical foundation. It is, at best, an interpretive stance on the manifold interactive phenomena of human caregiver-infant interactions. Importantly, for purposes of the present argument, these adaptationist approaches to understanding the development of joint attention in humans do not predict (a) the emotional features of the environmental contexts in which human signaling develop or (b) the cross-cultural variability displayed in the development of joint attention. As such, our finding of the pairing of positive emotional signals with referential gestures by adult caregivers neither confirms nor disconfirms an adaptationist interpretive stance; in other words, adaptationist theories are not falsifiable on the basis of our findings. Thus, the theoretical significance of our findings, in our view, is that we were able to test a key tenet of learning- and ecologically based environmental accounts of the development of joint attention in humans – that the environment must provide a differential reward structure – and the social learning approach survived this test of its predictions. Given the distribution of this gestural/affective synchrony in parental signaling across a very large range of infancy, future studies would add substantially to our understanding of the integration of emotional and referential signaling in the early lives of children. For example, this kind of analysis could be extended to infants’ home environments, like the seminal studies of Clarke-Stewart (1973, and see Rossmanith et al., in press). Moreover, future studies should explore the auditory/verbal concomitants of referential gestures in caregiver–infant interactions. Coding archival and future footage of parent-infant interactions in a range of cultural contexts could provide valuable insight into cross-cultural patterns of similarity and difference in affective/gestural synchrony, using these relatively simple measures of smiling and gestures. Hence, the essential finding of parents pairing their deictic gestures with smiles has significant relevance for theory development in this area. For example, the kind of affective/referential synchrony we report, here, throughout the infancy period, might complement the dynamic-gesture/word (visual/auditory) synchrony that figures prominently in Zukow-Goldring’s (1997) theory of attention-shaping through perception of amodal invariants.

With respect to the specific theoretical concerns of the present special issue, the present findings are consistent with environmentally situated accounts of child cognitive development. The episodes of joint attention that we elicited in the laboratory were encapsulated with positive affective expression, even though the parents received no instruction to do so. Their spontaneous display of positive emotion is consistent with Hobson’s (1993) postulate of affective bridges to conceptually based social awareness, a point of view that highlights the embodied, situated nature of infants’ developing social competencies. The contemporary practice of attributing developmental change solely to hypothetical, hidden changes in psychological processes can direct researchers’ attention away from the empirical, psychologically relevant bodily realities of human parent-infant engagement patterns (e.g., Shotter and Newson, 1982; Zukow-Goldring, 1997; Reddy, 2001, 2003; Leudar and Costall, 2004; Striano and Bertin, 2005; Leavens and Bard, 2011; Bard and Leavens, 2014). This study suggests that an increased awareness of the affective components of deictic communication may reveal the previously underappreciated and public information available not only to researchers but to parents and young children tasked with building routines of meaning.

## Conflict of Interest Statement

The authors declare that the research was conducted in the absence of any commercial or financial relationships that could be construed as a potential conflict of interest.
